# Case of Lithotomy

**Published:** 1849-05

**Authors:** Paul F. Eve

**Affiliations:** Professor of Surgery in the Medical College of Georgia


*Case of Lithotomy. One hundred and seventeen Calculi, weighing four and a half ounces, successfully removed.* By Paul F. Eve, M.D., Professor of Surgery in the Medical College of Georgia.

A brief notice of the following case, against the writer's expressed wish, was made in one of our newspapers. It is proposed to record it now, where, if it possesses sufficient interest, it legitimately belongs.

In the severe September gale of 1824, Mr. O'Bannon, then a lad of 18 years, was engaged at work upon a house, which was blown down. In the fall, he was struck upon the back with a piece of timber, and from the injury then received he dates his difficulty in urinating. During the twenty-four years he has been a sufferer, Mr. O'B. has fully tested the prescriptions of the *unprofessional* of several States, and he has traveled far and near in search of relief. He even became a sailor on the ocean; but all to no purpose, his disease continued to harrass him day and night.

For the past two years his difficulty became so great, that to discharge urine at all, he had to assume the horizontal position, and then with the fingers introduced into the rectum, he pushed up the bladder. A large quantity of matter, he says, is also evacuated by the penis. When he sits upon the edge of a chair he experiences a sensation as of crushing (crepitation) a ball of snow in the perineum.

In December he entered the charitable Institution under our Faculty, and a catheter was for the first time attempted to be introduced. This came at once in contact with a calculous mass in the perineum, where a tumor was found, projecting to the right of the raphe running back from the scrotum.

*Operation.*—On the 16th of last month (January) the following operation was performed in the presence of the Medical Class of our College—chloroform was administered by Dr. Means. After the patient was placed in the usual position for lithotomy, an incision, about three inches in length, was made over the tumor situated in the perineum, as for the lateral operation, except that it was upon the right, instead of the left side. About 56 calculi were removed through the opening, and it was hoped the operation was completed; but upon introducing a female catheter through the wound into the bladder, a second collection of stones was readily detected in this receptacle. A grooved sound was now passed through the urethra and the double lithotome conducted by it into the bladder; the former was withdrawn and the bi-lateral section completed, by drawing the latter instrument out somewhat in the line of the external incision made in the skin. With the lithotomy forceps repeatedly introduced, by conducting it upon the finger, 61 stones were extracted from the bladder. Through the opening in the perineum a quantity of pus was discharged. During the operation, the rectum protruded in a large mass so as to interfere with lowering the handle of the forceps, to seize the calculi in the bladder. The patient also had violent and involuntary contractions of the abdominal muscles, and during the latter stage of the operation the chloroform was discontinued. It lasted one hour. He was so reduced by his long suffering, a period of twenty-four years and four months, that after the operation I took him like a child in my arms and carried him up a flight of stairs to his room.

The following is the analysis of the calculus, kindly made by Professor Means, and addressed to me:

"The urinary calculus, taken from the bladder of Mr. O'Bannon, has been subjected, at your request, to a chemical analysis, and merits at my hand the following description, viz:

*External form.*—The particular calculus under consideration, is but a fair specimen, both in its physical properties and chemical constituents, to every other of the entire number removed from the perineum and cystic cavity of your recent patient, and which, by your courtesy, I was privileged to examine both during, and after the extraordinary operation. Being a solid, bounded by four oblique planes, it presents the tetrahedral shape distinctly; its solid angles and lateral edges, instead of being regularly truncated, and replaced by tangent planes, exhibit gently rounded surfaces, which gradually blend with the respective faces, and are evidently the result of constant attrition, kept up for many years.

*Physical characteristics.*—The exterior furnishes a beautifully smooth, and even polished surface. The structure is laminated with admirable parallelism—the respective tunics conforming to the external figure of the stone, and easily separable by the nail—the fracture uneven, and the powder, harsh and gravelly under the touch.

The predominant color is a greyish white, which is frequently substituted, however, in the more deeply seated laminæ, by a pale-brown tint. Its specific gravity, is 1.02.

*Chemical constituents.*—I had anticipated the uric acid calculus, but the use of the blow-pipe flame, and the application of appropriate acid and alkaline tests, soon revealed the presence of Phosphate of Lime almost pure. This form of urinary concretion has been pronounced by Silliman, Gardner and others, as very rare. It is peculiar, however, to the prostate gland, in the neighborhood of which the calculi, in your recent operation, were found to be imbedded, and which probably controlled the chemical affinities that subsequently deposited so large a mass in the fundus of the bladder. Its chemical elements are 3 atoms of Phosphoric Acid, 8 of Lime, and one of basic water, as expressed in the following formula:—$8CaO, HO+3PO_5$.

*The fusible Calculus* (Phosphate of Ammonia and Magnesia) has in one or two instances, reported in the Philosophical Transactions, for 1809, been found in such quantity as nearly to fill the cavity of the bladder, but so large a mass of Bone-earth calculi, is surely a still more rare occurrence.

The whole number extracted was 117, of which the largest weighed ʒij. and 38 grs.; the two next in size, each 78 grs., and the smallest 1 gr.—furnishing an aggregate weight of ℥ ivss."

As usual with me, no dressing was applied to the wound, but the patient was requested to keep his knees together and to remain perfectly quiet. He took 40 drops of laudanum the night after the operation, and his diet was restricted to cold lemonade flaxseed tea. He also omitted the medicines upon which he had been placed, viz., Peruvian bark and sulph. iron, with volatile alkali occasionally.

January 7th. Had passed a pretty good night. Some urine had even been already voided by the natural passage, notwithstanding the opening in the perineum. He has bathed himself in warm water; has now no fever, is quite cheerful, smokes his pipe, and has taken some soup, table tea, and an orange.

Jan. 8th. Is doing well. Has had a good night—the best, he says, for years past. Uses a bed-pan to prevent soiling the clothes. Has sat up a little by the fire.

He has continued gradually to improve, notwithstanding the unfavorable state of the weather. No other application to the wound than castile soap and warm water, several times daily.

On the 10th, four days after the operation, he changed his room. He experienced the next day some uneasiness in urinating, and had for a day or two slight diarrhea.

On the 17th, the 11th day since the operation, he was out in the yard walking about. By pressing the edges of the wound together he could now pass nearly all the urine through the urethra.

On the 24th of January, i. e., the 18th day after he was disembarrassed of his numerous calculi, Mr. O'Bannon returned home, a distance of 22 miles. The wound had nearly healed. He is to use, as a tonic, small doses of sulph. of quinine and iron.

A month after the operation, a special messenger reports him entirely well.

In noticing the peculiarities of this case, we remark, first, the cause—an injury to the spinal column, probably by partial paralysis of the bladder favoring a perversion of the function of this organ.

2d. The nature of the calculus—phosphate of lime or bone-earth. This is, I believe, peculiar to disease of the bladder itself. Any calculus may have a coating of phosphate of lime, but when composed throughout of this combination, the evidence is strong, if not conclusive, that the evil originated in the bladder.

3d. The long existence of the disease without its character being detected.

4th. The size and shape of the calculi. They appeared both in the perineum and bladder to have been regularly impacted, one against the other. Occasionally two, but generally one only was seized by the forceps in their extraction.

5th. The membranous portion of the urethra preserved its integrity, while the bulbous was ruptured by the stones. The two deposites, the one in the perineum and the other in the bladder, were about two inches apart.

6th. The calculi must have all had a common origin—there being no difference in their shape, color or composition. Those in the bladder were, however a little larger than those taken from the perineum. I agree with Prof. Means in the opinion that they probably originated in the prostate gland, observing the laws of chrystalization in their subsequent accumulation in the bladder and perineum.

7th. The remarkable fact that Mr. O'Bannon preserved his virile powers. His wife has borne several children, and is now actually seven months pregnant.

8th. The speedy recovery, in certainly, what must be considered, quite unfavorable circumstances.—*N. Orleans Med. and Surg. Jour.*

